# CEBPD Potentiates the Macrophage Inflammatory Response but CEBPD Knock-Out Macrophages Fail to Identify CEBPD-Dependent Pro-Inflammatory Transcriptional Programs

**DOI:** 10.3390/cells10092233

**Published:** 2021-08-28

**Authors:** C. Arnold Spek, Hella L. Aberson, Joe M. Butler, Alex F. de Vos, JanWillem Duitman

**Affiliations:** 1 Center for Experimental and Molecular Medicine, Amsterdam UMC, University of Amsterdam, 1105 AZ Amsterdam, The Netherlands; h.l.aberson@amsterdamumc.nl (H.L.A.); j.m.butler@amsterdamumc.nl (J.M.B.); a.f.devos@amsterdamumc.nl (A.F.d.V.); J.W.Duitman@amsterdamumc.nl (J.D.); 2 Department of Respiratory Medicine, Amsterdam UMC, University of Amsterdam, 1105 AZ Amsterdam, The Netherlands

**Keywords:** CCAAT/enhancer-binding protein delta, CEBPD, LPS, macrophage, inflammation

## Abstract

CCAAT/enhancer-binding protein delta (C/EBPδ) is a member of the C/EBP family of transcription factors. According to the current paradigm, C/EBPδ potentiates cytokine production and modulates macrophage function thereby enhancing the inflammatory response. Remarkably, however, C/EBPδ deficiency does not consistently lead to a reduction in Lipopolysaccharide (LPS)-induced cytokine production by macrophages. Here, we address this apparent discrepancy and show that the effect of C/EBPδ on cytokine production and macrophage function depends on both the macrophage subtype and the LPS concentration used. Using CRISPR-Cas generated macrophages in which the transactivation domain of C/EBPδ was deleted from the endogenous locus (ΔTAD macrophages), we next show that the context-dependent role of C/EBPδ in macrophage biology relies on compensatory transcriptional activity in the absence of C/EBPδ. We extend these findings by revealing a large discrepancy between transcriptional programs in C/EBPδ knock-out and C/EBPδ transactivation dead (ΔTAD) macrophages implying that compensatory mechanisms do not specifically modify C/EBPδ-dependent inflammatory responses but affect overall macrophage biology. Overall, these data imply that knock-out approaches are not suited for identifying the genuine transcriptional program regulated by C/EBPδ, and we suggest that this phenomenon applies for transcription factor families in general.

## 1. Introduction

CCAAT/enhancer-binding protein delta (C/EBPδ), also known as nuclear factor interleukin (IL)-6β, is a member of the C/EBP family of transcription factors containing six members, C/EBPα, C/EBPβ, C/EBPδ, C/EBPε, C/EBPγ and C/EBPζ [1,2]. All these members share substantial sequence identity in the C-terminal region, which consists of a basic amino-acid-rich DNA-binding domain followed by a leucine zipper imperative for homo- or hetero-dimerization and subsequent DNA binding. Due to the conserved nature of the C-terminal region, all C/EBP family members, except C/EBPζ, bind to identical DNA recognition sites in the promotor regions of C/EBP target genes [3]. The subsequent transcriptional activity is determined by transactivation domains present in the N-terminus of the C/EBP family members. Specific interactions between the transactivation domains and components of the basal transcription machinery ultimately dictate transcriptional activity that may range from strong activation to dominant negative inhibition by transactivation domain-less C/EBPγ.

According to the current paradigm, C/EBPδ acts as a pro-inflammatory transcription factor enhancing the expression of pro-inflammatory mediators [4,5]. Indeed, C/EBPδ is shown to regulate IL-1β or collagen-induced cyclo-oxygenase-2 (COX-2) expression [6,7,8]; to induce toll-like receptor (TLR)-4 expression and subsequent signalling [9]; to potentiate Fcγ receptor-mediated tumor necrosis factor alpha (TNFα), macrophage inflammatory protein (MIP)-2 and MIP-1α expression [10]; to mediate IgG immune complex-induced macrophages inflammatory cytokine and chemokine production in macrophages [11,12]; and to enhance lipopolysaccharide (LPS)-induced IL-6, monocyte chemoattractant protein-1 (MCP-1) and endothelin-1 levels [13]. In line with its proinflammatory role in vitro, C/EBPδ also seems to play an important role in the inflammatory response in vivo. C/EBPδ is suggested to drive LPS-induced systemic inflammation, as reflected by reduced TNFα and IL-6 levels in C/EBPδ deficient mice [14], whereas it also reported to induce TNFα, IL-6 and MIP-2 levels upon intranasal LPS inoculation [11]. Moreover, macrophage-dependent chemokine and cytokine expression is enhanced by C/EBPδ during collagen-induced rheumatoid arthritis [15]. Next to driving inflammation during sterile conditions, C/EBPδ also amplifies the inflammatory response during *Escherichia coli*-induced peritonitis [16]. This amplification is essential for bacterial elimination and survival as C/EBPδ deficient mice are more susceptible to the peritoneal infection as compared with wild-type animals. Similarly, C/EBPδ is expressed in infiltrating macrophages in the lung during *Klebsiella pneumoniae*-induced pneumonia and C/EBPδ deficiency enhanced pneumonia-induced mortality [17]. Overall, these data led to the current paradigm that C/EBPδ is an important driver of the inflammatory response both during sterile inflammation and during infectious disease.

Several data are at odds with the paradigm that C/EBPδ drives pro-inflammatory gene expression, however. Indeed, C/EBPδ deficiency does not limit LPS-induced cytokine production (i.e., TNFα, MCP-1 and IL-6) by ex vivo stimulated bone marrow-derived macrophages [17,18]. Moreover, cytokine levels are even increased in the plasma of C/EBPδ deficient mice during *Klebsiella pneumoniae*-induced pneumonia [17] and in the bronchoalveolar lavage fluid (BALF) of LPS-treated C/EBPδ deficient mice [19].

The experiments supporting the current paradigm as well as those at odds with a pro-inflammatory role of C/EBPδ all used knock-out cells and/or mice. Although such model systems are well-established and contribute significantly to unravelling the (patho)physiological role of many proteins, one could question the validity of such an approach for transcription factors with overlapping DNA recognition sites. Indeed, deletion of a single transcription factor (i.e., C/EBPδ in this case) allows binding of an alternative transcription factor with unforeseen outcomes. If the alternative transcription factor does not (or reduces the ability to) activate transcription of the gene of interest, expression of the target gene is reduced and it is (rightfully) concluded that C/EBPδ drives transcription of this target gene. If the alternative transcription factor is similarly effective in inducing transcription, deletion of C/EBPδ does not change expression levels, and one would (wrongfully) conclude that the gene of interest is C/EBPδ independent. In the case that the alternative transcription factor is an even more potent inducer of transcription than C/EBPδ, gene expression levels will increase due to C/EBPδ deletion, and one would (wrongfully) conclude that C/EBPδ inhibits expression of the gene of interest. It is worth noting that this is not merely a theoretic probability because certain gene promotors have already been shown to be regulated differentially by specific C/EBP family members in cultured cells [20,21,22], and redundancy between different family members has indeed been shown before [18,23].

In the current manuscript, we first address the context-dependent role of C/EBPδ in macrophage-dependent cytokine expression. Next, we assess whether the observed differences could indeed be explained by compensatory mechanisms dependent on alternative transcription factor binding in C/EBPδ knock-out macrophages by comparing LPS-induced cytokine production in C/EBPδ knock-out and C/EBPδ “transcription dead” macrophages.

## 2. Materials and Methods

### 2.1. Animals

C/EBPδ deficient mice, generated as described previously [24], were backcrossed at least 10 times to a C57BL/6 background. C57BL/6 wild-type mice were purchased from Charles River. Animals were maintained at the animal facility of the Academic Medical Center (University of Amsterdam) with free access to food and water. All animal experiments were approved by the Institutional Animal Care and Use Committee of the Academic Medical Center, University of Amsterdam.

### 2.2. Bone Marrow Derived Macrophage (BMDM) Isolation

BMDMs were isolated from C/EBPδ deficient and wild-type mice, as described before [17]. In detail, tibia and femur of naïve wild-type and C/EBPδ^−/−^ mice were harvested, and bone marrow was flushed out of the bones with RPMI 1640 supplemented with 10% fetal calf serum, 2 mM L-glutamine, 100 units/mL penicillin and 500 μg/mL streptomycin (all Lonza, Basel, Switzerland). Erythrocytes were lysed by incubation in erythrocyte lysis buffer for 10 minutes at room temperature after which the remaining cells were plated in a non-tissue culture treated petri dish in complete RPMI medium containing 30 ng/mL M-CSF (Macrophage-colony stimulating factor; CYT-43; ProSpec, Rehovot, Israel) in order to allow macrophage differentiation. After 7 days, cells were harvested using 4 mg/mL lidocaine in 5 mM EDTA/PBS and immediately used for stimulation experiments.

### 2.3. Peritoneal Macrophage Isolation

Peritoneal lavage cells were obtained from C/EBPδ deficient and wild-type mice by flushing the peritoneal cavity with ice-cold sterile PBS, as described before [25]. Peritoneal cells were washed, counted, resuspended in RPMI 1640 medium containing 10% fetal calf serum, 2 mM L-glutamine, penicillin/streptomycin and incubated in 96-well flat-bottom microtiter plates (1 × 10^5^ cells in 100 mL/well) at 37 °C with 5% CO_2_ for 2 h. Finally, adherent macrophages were washed with culture medium to remove non-adherent cells after which the cells were immediately used for stimulation experiments.

### 2.4. Cell Stimulation Experiments

Primary macrophages (10,000) seeded in 100 μL in 96-wells plates were, upon adherence of the cells, stimulated with 100, 1, 0.1 or 0 ng/mL LPS (L4268, Sigma-Aldrich, Saint Louis, MO, USA) for 24 h after which the supernatant was collected and stored at −20 °C for further analysis. The cells were subsequently lysed using TriReagent (#11667165001; Roche Diagnostics, Basel, Switzerland) for mRNA isolation. Cells of 2 wells were pooled in order to obtain sufficient amounts of mRNA for further analysis.

### 2.5. Cytokine Measurements

TNFα, IL-6 and MCP-1 levels were determined using a cytometric beads array multiplex assay (BD Biosciences) essentially as described before [26].

### 2.6. RNA Isolation, cDNA Synthesis and RT-qPCR

RNA was extracted form TriReagent lysed cells according to routine procedures. Eluted RNA was analyzed spectrophotometrically using a NanoDrop 2000 (Thermo Fisher Scientific, Breda, The Netherlands). All samples were treated with RQ1 RNAse-Free DNAse (Promega Benelux BV, Leiden, The Netherlands) and reverse-transcribed into cDNA using M-MLV Reverse Transcriptase (Promega Benelux BV), random hexamers (Fisher Scientific) and 10 mM dNTPs (Fermentas, Waltham, MA, USA). The SensiFAST™ SYBR^®^ No-ROX Kit (GC biotech, Waddinxveen, The Netherlands) was used to perform real-time quantitative RT-PCR on a LightCycler^®^ 480 Instrument II (Roche Molecular Systems, Rotkreuz, Switzerland) using the primers listed in Supplementary Table S1.

### 2.7. Mining of Publicly Available RNA Microarray Datasets

Datasets were derived from Gene Expression Omnibus [27] using the R2 microarray analysis and visualization platform [28]. *CEBPA*, *CEBPB*, *CEBPD*, *CEBPG*, *CEBPE* and *CEBPZ* expression levels were derived from two different datasets, i.e., GSE5099 [29] and GSE46903 [30]. From the GSE46903 dataset, we only included data from stimulations for 24–72 h.

### 2.8. CRISPR/Cas9 Genome Editing

Homozygous knock-out and transactivation mutant (ΔTAD) C/EBPδ RAW264.7 macrophages were generated using CRISPR/Cas9 technology according to the protocol of Ran et al. [31]. Using the crispr.mit.edu online tool, different single-guide RNAs (sgRNAs) were designed to target the sequence upstream (5′ guides; before amino acid 40) or downstream (3′ guides; after amino acid 108) of the transactivation domain of C/EBPδ (Supplemental Figure S1). 5′ guides (CEBPD_sgRNA1: 5′-GAGGGTGGACAAGCCCGGCCG-3′; CEBPD_sgRNA2: 5′-GCCGGCCGAGGGCCCGAGCCA-3′ and CEBPD_sgRNA3: 5′-GGATCCCCTGGCTCGGGCCCT-3′) were cloned into the pSpCas9 (BB)-2A-GFP (PX458) vector (Addgene; plasmid 48138), whereas 3′ guides (CEBPD_sgRNA4: 5′-GCTACGCGACCCCCGGGTGTG-3′ and CEBPD_sgRNA4: 5′-GGGCGGCCCTACGCGACCCCC-3′) were cloned into the pSpCas9 (BB)-2A-Puro (PX459) vector (Addgene; plasmid 48139). RAW264.7 cells were transfected with different combinations of 5′ (P458) and 3′ (PX459) guide plasmids using jetPEI transfection reagent (Westburg). Forty-eight hours post transfection, GFP positive cells were sorted using FACSAria (BD Biosciences), and single cells were subjected to puromycin selection. After expansion, cells were collected for PCR analysis using primers spanning the transactivation domain (Fw: 5′-CCTTCTACGAGCCAGGCAGG-3′; Rv: 5′-GCCCTTTTCTCGGACTGTG-3′), and selected clones were confirmed using genotype-specific forward primers (knock out: 5′-CCCGAGTGTGGGGTCTGT-3′ and TAD: 5′-GAGGCCCGGGTGTGG-3′). Finally, potential *C/EBPδ* ΔTAD clones were validated by sequencing the genomic DNA region around the transactivation domain using BigDye terminator (Thermo Fischer). Out of frame ΔTAD mutant clones obtained during the CRISPR approach were used as full knock-out cells (Supplemental Figure S2).

### 2.9. RAW264.7 Cell Culture and Stimulation

Murine RAW264.7 cells (TIB-71; ATCC) and all engineered isogenic variants (CRISP/Cas9-derived *C/EBPδ* knock-out and ΔTAD RAW264.7 cells) were cultured in IMDM medium (Gibco), supplemented with 10% fetal calf serum, 2 mM L-glutamine, 100 units/mL penicillin and 500 μg/mL streptomycin (all Lonza). Cells were maintained in a humidified incubator at 37 °C, 5% CO_2_ and 95% air. For stimulation experiments, cells (17,500) were seeded in 100 μL in 96-wells plates supplemented with 100, 1, 0.1 or 0 ng/mL LPS (L4268, Sigma) for 24 h after which the supernatant was collected and stored at −20 °C for further analysis. The cells were subsequently lysed using TriReagent (#11667165001; Roche Diagnostics) for mRNA isolation. Cells of 2 wells were pooled in order to obtain sufficient amounts of mRNA for further analysis.

### 2.10. RNA Library Preparation and Sequencing

Total RNA was isolated from wild-type, C/EBPδ knock-out and ΔTAD RAW cells using the NucleoSpin RNA miniprep kit (BIOKÉ, Leiden, The Netherlands). RNA quality was assessed by bioanalysis (Agilent Technologies, Santa Clara, CA, USA), with all samples having RNA integrity numbers (RINs) >7. Total RNA concentrations were determined by Qubit^®^ 2.0 Fluorometer (Life Technologies, Carlsbad, CA, USA). Sequencing libraries were prepared by means of the KAPA RNA HyperPrep with RiboErase (Roche Diagnostics) as per manufacturer’s instructions. Libraries were sequenced using the Illumina HiSeq4000 (Illumina, San Diego, CA, USA) to generate 50 bp single-end reads.

### 2.11. Bioinformatics Analysis of RNAseq

The sequence read quality was assessed using FastQC methods (version 0.11.5; Babraham Institute, Babraham, UK). Trimmomatic version 0.39 [32] was used to trim the Illumina adapters and filter low quality reads and ambiguous nucleotide-containing sequences. Low quality leading (3 nucleotides) and trailing (3 nucleotides) bases were removed from each read. A sliding window trimming using a window of 4 and a phred score threshold of 15 nucleotides was used to access the quality of the reads. After pre-processing, the remaining high-quality reads were aligned against the mouse Genome Reference Consortium Build 38 (GRCm38, Ensembl release 101) using Hisat2 (version 2.2.0; University of Texas Southwestern Medical Center, Dallas, TX, USA) [33] with default parameters. Count data were generated by means of the HTSeq method [34], and differential gene expression was analyzed using the R2 microarray analysis and visualization platform [28] with a false discovery adjusted *p*-value of less than 0.01. To analyze the function of the differentially expressed genes, Kyoto Encyclopedia of Genes and Genomes (KEGG) pathway enrichment analyses were conducted with a *p*-value cut-off of less than 0.005.

### 2.12. Statistical Analysis

All data are expressed as means ± SEM. Differences between groups were analyzed by one-way ANOVA with Bonferroni correction for multiple testing. Analyses were performed using GraphPad Prism version 8 (GraphPad Software, San Diego, CA, USA). Statistically significant differences were considered with a *p*-value of less than 0.05.

## 3. Results

### 3.1. Context-Dependent Effect of C/EBPδ on LPS-Induced Cytokine Production

**Figure 1:**
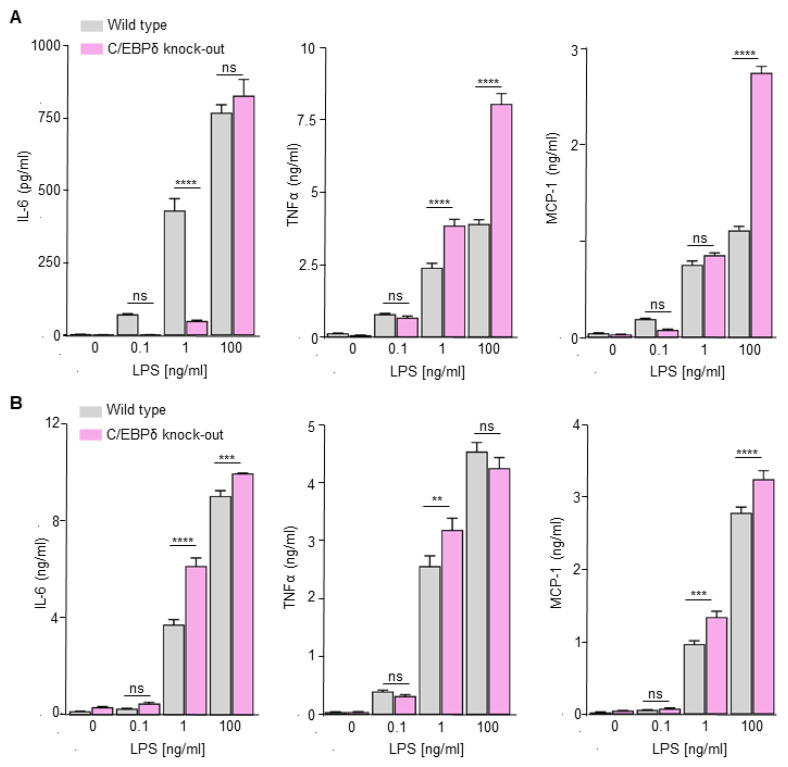
LPS-induced cytokine production in wild-type and C/EBPδ knock-out M-CSF differentiated BMDMs (**A**) and peritoneal macrophages (**B**). Macrophages from wild-type or C/EBPδ knock-out mice were stimulated with different LPS concentrations for 24 h after which TNFα, MCP-1 and IL-6 levels were analyzed. Shown is the mean ± SEM (*n* = 6–8) of a representative experiment performed in duplo. **: *p* < 0.01; ***: *p* < 0.001; ****: *p* < 0.0001; ns: non-significant.

At odds with the general notion that C/EBPδ potentiates TNFα, IL-6 and MCP-1 expression by macrophages [13,15,16,35], we previously showed that loss of C/EBPδ did not reduce cytokine levels produced by LPS-stimulated BMDMs [17]. To provide more insight into the apparent complex context-dependent role of C/EBPδ in pro-inflammatory cytokine production, we stimulated M-CSF differentiated macrophages and primary peritoneal macrophages with different LPS concentrations. As shown in Figure 1, LPS induced a dose-dependent increase in IL-6, TNFα and MCP-1 in both wild-type M-CSF differentiated BMDMs (Figure 1A) and peritoneal macrophages (Figure 1B). It is worth noting that C/EBPδ deficiency affected LPS-induced cytokine production in concentration and cell type dependent manner. Indeed, IL-6 levels were reduced in M-CSF differentiated C/EBPδ knock-out BMDMs at the low (although not significant) and intermediate, but not high, LPS concentration. MCP-1 levels were reduced in M-CSF differentiated C/EBPδ knock-out BMDMs stimulated with low dose LPS, not affected by the intermediate LPS dose and increased after high dose LPS stimulation. TNFα levels were similar in M-CSF differentiated C/EBPδ knock-out BMDMs at the low LPS concentration but increased in C/EBPδ knock-out BMDMs at the intermediate and high dose LPS. In contrast to BMDMs, none of the conditions analyzed resulted in reduced cytokine production by C/EBPδ knock-out peritoneal macrophages. As shown in Figure 1B, LPS-induced cytokine production was largely similar in wild-type and C/EBPδ knock-out peritoneal macrophages at all LPS concentrations analyzed, with some small increased levels in knock-out macrophages at the intermediate or high LPS concentration. It thus seems that the effect of C/EBPδ on cytokine production by macrophages is both dependent on the macrophage subtype and the LPS concentration used.

### 3.2. Macrophage Differentiation/Activation Modifies Expression Levels of Alternative CCAATT Box Binding Proteins

**Figure 2:**
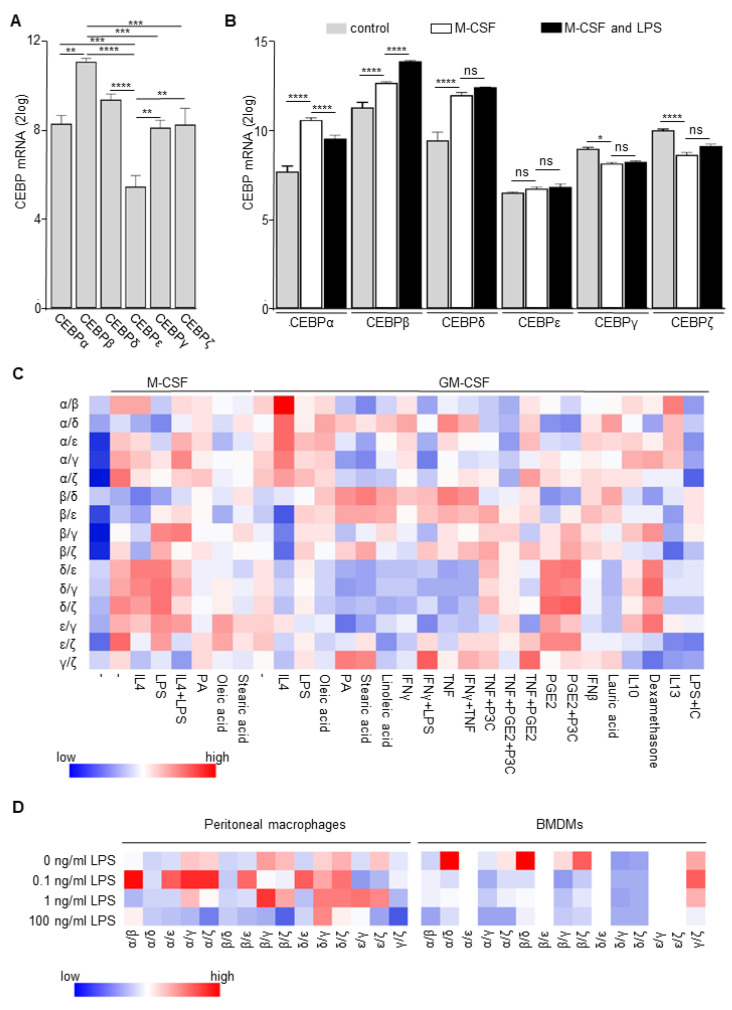
CEBP family member expression in monocytes and macrophages. (**A**) C/EBPα, C/EBPβ, C/EBPδ, C/EBP, C/EBPγ and C/EBPζ mRNA expression in freshly isolated human monocytes derived from GSE5099 and GSE46903. Mean ± SEM (*n* = 3); **: *p* < 0.01; ***: *p* < 0.001; ****: *p* < 0.0001. (**B**) C/EBPα, C/EBPβ, C/EBPδ, C/EBPε, C/EBPγ and C/EBPζ mRNA expression in naïve, M-CSF and M-CSF/LPS treated monocytes from GSE46903. Mean ± SEM (*n* = 3–5). *: *p* < 0.05; ****: *p* < 0.0001; ns: non-significant. (**C**) Heatmap of the expression ratio of C/EBP family members in monocytes differentiated and activated with different stimuli (data derived from GSE46903). (**D**) Heatmap of the expression ratio of C/EBP family members in BMDM and peritoneal macrophages stimulated with different LPS concentrations (*n* = 3–4). Note that C/EBPε levels were below the detection limit in the BMDM samples.

Next to C/EBPδ, several other transcription factors bind to CCAAT containing DNA recognition sites [36] and the absence of C/EBPδ allows these transcription factors to bind to promoter regions normally occupied by C/EBPδ. Depending on the relative transcriptional activity of these alternative transcription factors, binding could either reduce or enhance transcriptional activity. To address whether such a scenario could potentially explain the observed context-dependent effect of C/EBPδ deficiency on cytokine production, we first assessed the expression pattern of the C/EBP family members in primary monocytes. As shown in Figure 2A, all C/EBP family members are expressed in patient-derived primary monocytes with the highest levels observed for C/EBPβ and the lowest levels for C/EBPε. Interestingly, M-CSF-dependent monocyte differentiation leads to increased levels of C/EBPα, C/EBPβ and C/EBPδ and slightly diminished levels of C/EBPγ and C/EBPζ (Figure 2B). Subsequently, LPS stimulation further enhances the levels of C/EBPβ, reduces C/EBPα levels and does not affect the expression levels of the other C/EBP family members (Figure 2B). Monocyte-macrophage differentiation and subsequent activation thus seems to modify the relative expression levels of the individual C/EBP family members, and the ratios do indeed vary largely dependent on the differentiation/activation status (Figure 2C). To substantiate these findings, we next determined expression levels of the C/EBP family members in the BMDM and peritoneal macrophages stimulated with different LPS concentrations shown in Figure 1. Again, relative expression levels of the individual C/EBP family members vary across the experimental conditions resulting in different ratios (Figure 2D). It is worth noting that C/EBP family members are not the only transcription factors that bind to CCAAT box containing DNA recognition sites. Among others, the nuclear transcription factor Y (NF-Y) family also binds to the CCAAT recognition site [37], and the NF-Y family members may consequently also compensate for the loss of C/EBPδ. All three NF-Y family members are expressed in monocytes, and monocyte-macrophage differentiation/activation differentially affects C/EBP/NF-Y ratios (Supplemental Figure S3). Overall, we thus show that expression levels of transcription factors that may bind to the C/EBPδ recognition site in knock-out macrophages vary in a context-dependent manner, and we suggest that this could explain the discrepant results of C/EBPδ deficiency in cytokine production.

### 3.3. Diminished LPS-Induced Cytokine Production in C/EBPδ Transactivation Domain Mutant Macrophages

**Figure 3:**
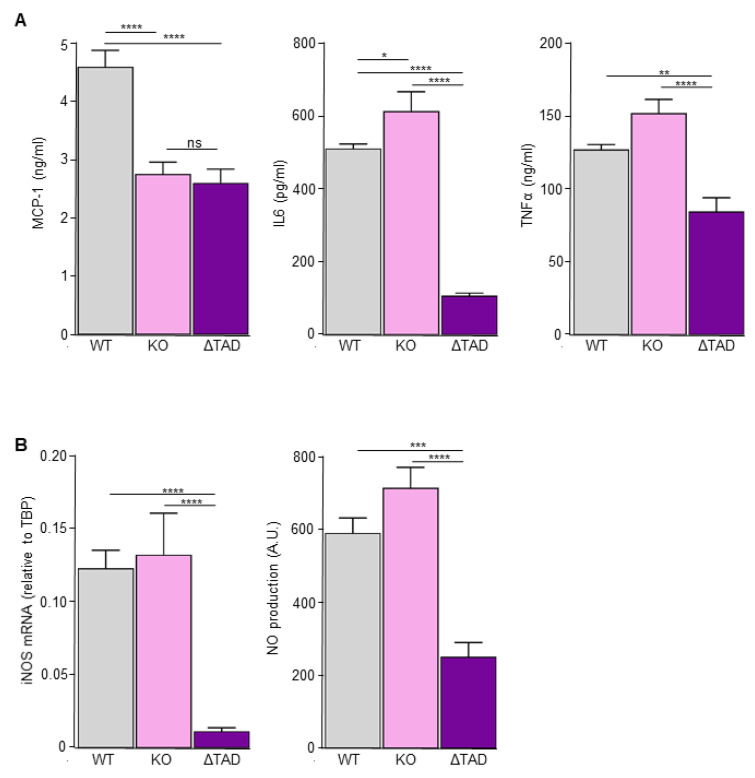
LPS-induced inflammatory and antibacterial response in wild-type (WT), C/EBPδ knock-out (KO) and C/EBPδ TAD mutant (ΔTAD) macrophages. (**A**) MCP-1, IL-6 and TNFα levels determined by ELISA in the supernatant of RAW264.7 macrophages stimulated with 100 ng/mL LPS for 24 h. (**B**) iNOS mRNA expression (left panel) and NO production (right panel) by RAW264.7 macrophages stimulated with 100 ng/mL LPS for 24 h. Shown is the mean ± SEM of three independent experiments performed with two individual wild-type, ΔTAD and KO clones. *: *p* < 0.05; **: *p* < 0.01; ***: *p* < 0.001; ****: *p* < 0.0001.

To address the hypothesis that the context-dependent effect of C/EBPδ depends on compensatory mechanisms in knock-out cells, we generated a model system that precludes compensatory activity of alternative transcription factors. Specifically, we generated macrophages expressing endogenous levels of a C/EBPδ mutant with normal DNA binding activity but without a functional transactivation domain (ΔTAD; Supplemental Figure S1). These mutant cells that express ΔTAD-C/EBPδ under activity of its own promoter were subsequently used, in combination with complete knock-out and wild-type macrophages, to analyze LPS-induced cytokine expression. As shown in Figure 3A, LPS-induced MCP-1 levels are reduced in knock-out macrophages compared with wild-type macrophages, whereas both IL-6 and TNFα levels are slightly increased in knock-out macrophages compared with wild-type macrophages. Importantly, LPS stimulation of ΔTAD-C/EBPδ macrophages shows a completely different picture. Indeed, LPS-induced expression of all three cytokines is largely reduced in the ΔTAD macrophages.

C/EBPδ is suggested to be essential in the polarization of macrophages into antibacterial M1 macrophages [38]. Consequently, we next analyzed iNOS expression and nitric oxide (NO) production by wild-type, knock-out and ΔTAD macrophages. Both iNOS and NO levels were undetectable under unstimulated conditions (data not shown) and considerably increased after LPS stimulation (Figure 3B). Interestingly, LPS-induced iNOS and NO levels were similar in wild-type and knock-out cells but largely diminished in ΔTAD macrophages. Overall, a picture emerged that the inflammatory/bactericidal response was largely similar in wild-type and knock-out macrophages but significantly decreased in ΔTAD macrophages.

### 3.4. Discordant Transcriptional Programs in C/EBPδ Deficient and C/EBPδ Transactivation Domain Mutant Macrophages

**Figure 4:**
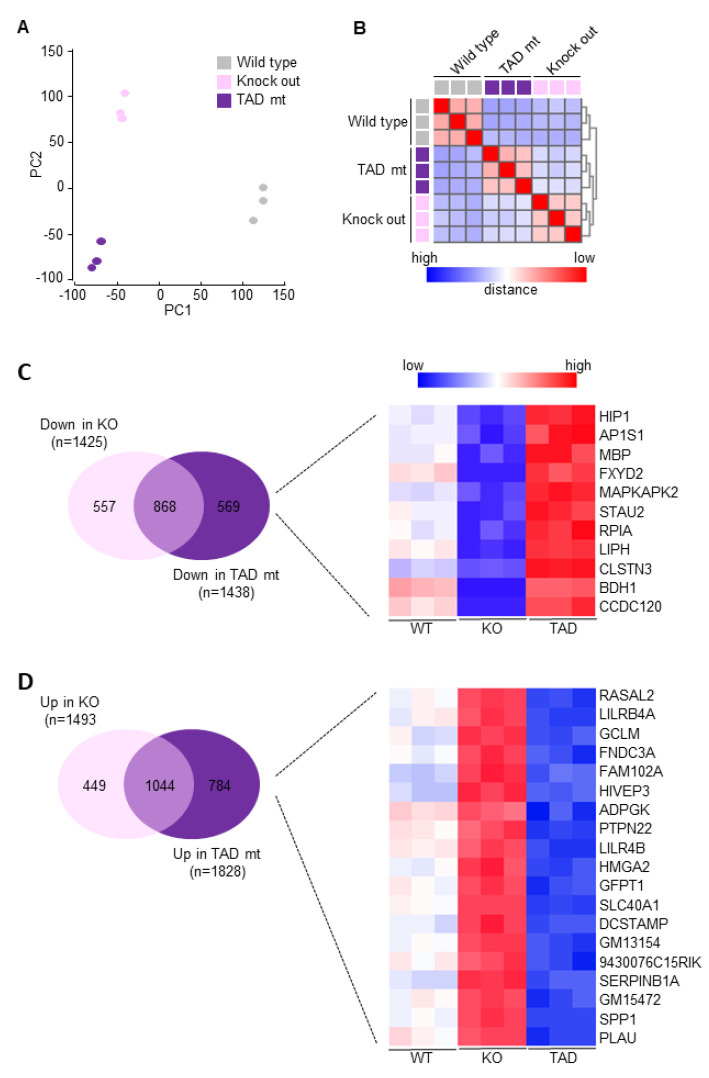
RNAseq gene expression analysis of wild-type (WT), C/EBPδ knock-out (KO) and C/EBPδ mutant (ΔTAD) macrophages. (**A**) Principal component analysis of pairwise genetic distances between the different macrophage subtypes. (**B**) Correlation heat map showing the Euclidean distances between the different macrophage subtypes. (**C**) Venn diagram showing overlap in downregulation of genes in knock-out and ΔTAD macrophages with a heatmap of genes downregulated in knock-out macrophages but upregulated in ΔTAD macrophages. (**D**) Venn diagram showing overlap in upregulation of genes in knock-out and ΔTAD macrophages with a heatmap of genes upregulated in knock-out macrophages but downregulated in ΔTAD macrophages. Heatmaps were generated using heatmapper (http://www.heatmapper.ca [39] assessed on 24 November 2020).

**Figure 5:**
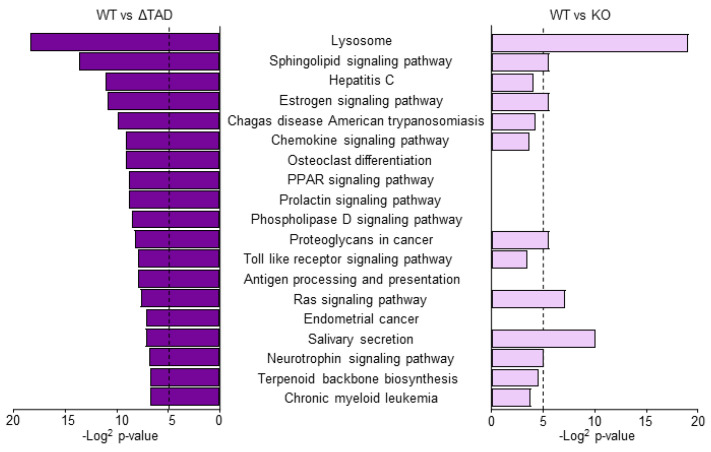
Functional enrichment analysis of differentially expressed genes. Significance (*p*-values) for KEGG pathways enriched in differentially expressed genes between wild-type and ΔTAD macrophages in wild-type vs. ΔTAD (**left**) and wild-type vs. knock-out (**right**) macrophages. The dotted lines indicate *p* = 0.05.

To fully appreciate the difference in gene expression between C/EBPδ knock-out and ΔTAD macrophages, we next performed RNAseq analysis on unstimulated naïve wild-type, knock-out and ΔTAD macrophages. Principal component analysis shows that the different macrophage subsets form well-separated clusters with no overlap between knock-out and ΔTAD macrophages (Figure 4A). The correlation heatmap shown in Figure 4B confirms the divergent gene expression patterns between the macrophage subsets, but it also suggests that C/EBPδ knock-out and ΔTAD macrophages are somewhat more strongly correlated with each other than with wild-type macrophages. On the individual gene level, a similar number of genes is downregulated in C/EBPδ knock-out and ΔTAD macrophages (i.e., 1425 and 1438, respectively). Of note, however, is that only approximately 60% (868) of the genes downregulated in C/EBPδ knock-out macrophages are also downregulated in ΔTAD macrophages. Of the remaining 557 genes downregulated in C/EBPδ knock-out macrophages, 546 genes are similar in wild-type and ΔTAD macrophages whereas 11 genes are even upregulated in ΔTAD macrophages compared to wild-type macrophages (Figure 4C). Similarly, a more or less equal number of genes is upregulated in both knock-out and ΔTAD macrophages (i.e., 1493 and 1828, respectively) but again only around 60% of the genes is upregulated in both genotypes, and 19 genes that are upregulated in knock-out macrophages are downregulated in ΔTAD macrophages (Figure 4D). The large difference in gene expression patterns between knock-out and ΔTAD macrophages also affects functional enrichment analysis of differentially expressed genes. Indeed, only 5 of the 19 KEGG pathways enriched in differentially expressed genes between wild-type and ΔTAD macrophages are also significantly enriched in differentially expressed genes between wild-type and knock-out macrophages (Figure 5). These RNAseq data thus show that transcriptional programs in C/EBPδ transactivation dead (ΔTAD) macrophages are largely different from those in full-blown C/EBPδ deficient (knock-out) macrophages.

## 4. Discussion

C/EBPδ is a member of the C/EBP family of transcription factors that, according to the current paradigm, potentiates cytokine production and modulates macrophage function, thereby enhancing the inflammatory response [5,22]. In the current manuscript, we show, however, that cytokine levels are not consistently reduced in LPS-stimulated C/EBPδ deficient macrophages. Actually, cytokine levels are only reduced in BMDMs stimulated with low LPS concentrations but not in peritoneal macrophages and BMDMs stimulated with high(er) LPS concentrations. We hypothesized that compensatory transcriptional activity of alternative CCAAT box binding transcription factors, which bind to promotor regions in knock-out cells that are normally occupied by C/EBPδ, could explain the context-dependent role of C/EBPδ in cytokine production. We provide evidence supporting this hypothesis by showing that LPS-induced cytokine levels are consistently reduced in ΔTAD macrophages.

We deleted the transactivation domain of CEBPD from its endogenous locus to generate ΔTAD macrophages. Such an approach ensures that mutant C/EBPδ expression levels in ΔTAD macrophages are similar to wild-type C/EBPδ levels in control cells. Moreover, mutant C/EBPδ binds to the same target gene promoters in ΔTAD macrophages as wild-type C/EBPδ does in control cells. By contrast, however, the absence of C/EBPδ in knock-out cells allows alternative CCAAT box binding factors to bind promoter regions occupied by C/EBPδ in control cells. Data interpretation is clearly obscured in such knock-out cells: indeed, if binding of the alternative transcription factor does not activate (or less efficiently activates) transcription of the gene of interest, expression of the target gene is reduced, and it is (rightfully) concluded that C/EBPδ drives transcription of this target gene. If the alternative transcription factor is similarly effective in inducing transcription, deletion of C/EBPδ does not change expression levels, and one would (wrongfully) conclude that the gene of interest is C/EBPδ independent. In the case that binding of the alternative transcription factor is more potent in inducing transcription, gene expression levels will increase due to C/EBPδ deletion, and one would (wrongfully) conclude that C/EBPδ inhibits expression of the gene of interest. Of note is that this is not merely a theoretic probability, because certain gene promotors have already been shown to be regulated differentially by specific C/EBP family members [20,21]. For instance, the nerve growth factor promotor is transactivated by C/EBPδ but not by C/EBPβ, although both family members display similar DNA-binding [21], whereas the Furin promotor is transactivated by C/EBPβ but not by C/EBPα or C/EBPδ [20].

C/EBPδ is not the only C/EBP family member for which knock-out experiments show surprising results. Ablation of C/EBPβ, which is generally believed to drive pro-inflammatory gene expression, unexpectedly increased serum IL-6 levels in mice upon *Candida albicans* inoculation [40] and did not affect expression levels of cytokines involved in macrophage activation, such as TNFα and IFNγ, in response to *Listeria monocytogenes* infection [41]. Moreover, ex vivo stimulations of wild-type and C/EBPβ deficient macrophages showed an expected diminished induction of IL-1β, TNFα, IL-6 and iNOS expression but an increased expression of IL-12 p40, RANTES and MIP-1β [42].

An elegant study of Lu and colleagues [18] suggests that C/EBPβ could compensate for the loss of C/EBPδ under certain (experimental) conditions. Indeed, LPS-induced IL-6 and TNFα levels are similar in wild-type and C/EBPδ deficient fetal liver-derived macrophages but are consistently reduced in C/EBPδ/C/EBPβ double-knock-out macrophages. Next to C/EBPβ, any C/EBP family member could obviously compensate for the loss of C/EBPδ and, considering the large variety in C/EBP ratios dependent on macrophage differentiation/activation, the compensating factor is likely context-dependent. Of note is that transcription factor binding to the C/EBP binding site is not limited to C/EBP family members. Indeed, the human immunodeficiency virus type 1 promoter contains overlapping binding sites for C/EBP and nuclear factor of activated T-cells (NFAT) and preventing NFAT nuclear localization resulted in enhanced C/EBPα binding [43]. Moreover, the CCAAT box is one of the most common cis-acting elements found in the promoter regions of a large number of genes [36], and many DNA binding proteins, for instance, NF-Y, interact with this sequence [37]. In knock-out cells, multiple transcription factors can thus bind to promotor regions that are normally occupied by C/EBPδ, which could explain the context-dependent effect of C/EBPδ deficiency.

We reveal a large discrepancy between transcriptional programs in C/EBPδ knock-out and C/EBPδ transactivation dead (ΔTAD) macrophages based upon which we advocate that knock-out approaches are not suited for identifying the genuine transcriptional program regulated by the transcription factor of interest (in this instance, C/EBPδ). This is likely not specific for C/EBPδ, as many transcription factors, such as the already mentioned NF-Y and NFAT families, but also the activator protein (AP)-1, AP-2 and Nuclear factor kappa B family members, bind to similar and/or overlapping recognition sites. Because most proteins are grouped in families [44], we envision that our conclusions are not specific for transcription factors but may actually extend to all studies that use knock-out conditions to identify gene expression patterns driven by the protein of interest, and thus, such results should be interpreted with care.

We showed that gene expression analysis of C/EBPδ knock-out macrophages does not accurately identify genes regulated by C/EBPδ. Despite this obvious limitation of knock-out studies for the identification of gene expression patterns by individual family members, gene knock-out approaches are highly relevant in biomedical research to identify (pre)clinical targets. Using C/EBPδ deficient animals, it has for instance been shown that C/EBPδ ablation protects against pneumococcal meningitis [45,46] and pneumonia [47], LPS-induced acute lung injury [11] and radiation-induced intestinal injury [48,49]. By contrast, C/EBPδ deficient mice are more susceptible to *Escherichia coli*-induced peritonitis [16] and *Klebsiella pneumoniae*-induced pneumonia [17], whereas breast tumor growth [50] and renal fibrosis [51] are also increased in C/EBPδ deficient mice. Although the underlying mechanism responsible for the observed differences in C/EBPδ deficient mice (i.e., abolishment of C/EBPδ driven transcription or increased compensatory mechanisms by alternative CCAAT box binding transcription factors) remain elusive, these studies identify C/EBPδ as a target to pursue in the former but not the latter life-threatening disorders.

## 5. Conclusions

C/EBPδ potentiates LPS-induced cytokine production, but this effect is obscured in C/EBPδ knock-out macrophages likely due to compensatory transcriptional programs induced by alternative CCAAT box binding factors that are expressed at normal levels. Our data imply that knock-out approaches are not suited for identifying the genuine transcriptional program regulated by C/EBPδ, and conclusions on the specific role of C/EBPδ have to be drawn with care. We envision this is not C/EBPδ specific but holds true for protein family members in general.

## Data Availability

All relevant data are within the paper and its Supporting Information files. Sequence libraries are publicly available through the National Center for Biotechnology Information gene expression omnibus under the following accession number: GSE173552.

## Supplementary Materials

The following are available online at https://www.mdpi.com/article/10.3390/cells10092233/s1, Figure S1: CRISPR-mediated generation of C/EBPδ ΔTAD mutants. Figure S2: DNA sequence of wild-type, C/EBPδ knock-out and C/EBPδ ΔTAD RAW264.7 clones, Figure S3: Relative CEBP and NF-Y family member expression in monocytes and macrophages, Table S1: Primer sequences used to assess C/EBP family expression.
