# Supplementary material for: CEBPD Potentiates the Macrophage Inflammatory Response but CEBPD Knock-Out Macrophages Fail to Identify CEBPD-Dependent Pro-Inflammatory Transcriptional Programs
Source: Cells. 2021 Aug 28;10(9):2233. doi: 10.3390/cells10092233 (PMC8470509; doi:10.3390/cells10092233)
Supplement: Supplementary file 1 [file cells-10-02233-s001.zip › cells-1312374-supplementary.pdf]

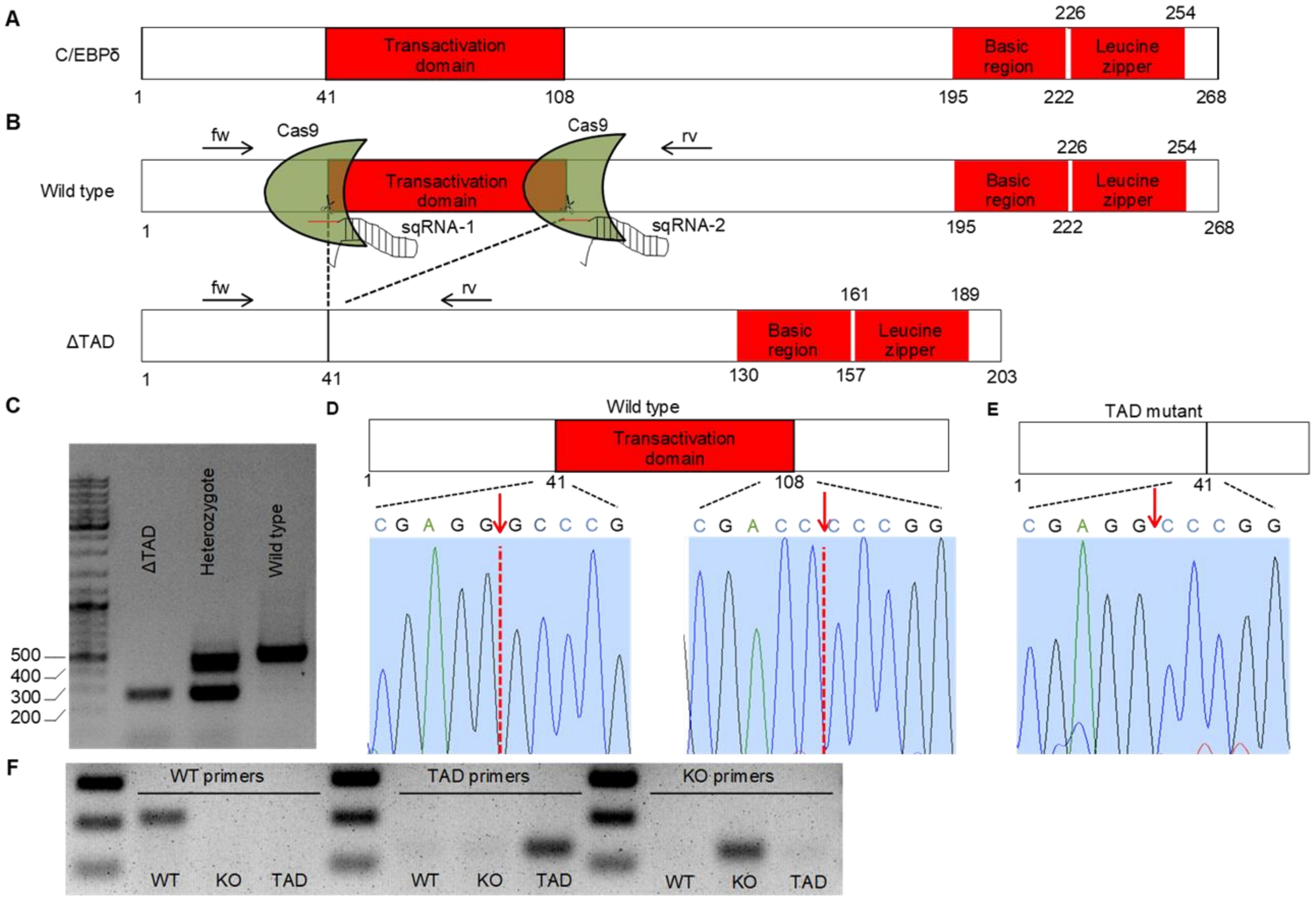

Figure S1: CRISPR-mediated generation of a C/EBPδ TAD mutant. (A) Overview of C/EBPδ showing the transactivation domain; the basic DNA binding region and the Leucine zipper. (B) Guide RNAs binding to opposite sides of the TAD results in CAS9 dependent deletion of the region between the guides. (C) PCR analysis of DNA isolated from RAW cells after CRISPR-mediated TAD deletion. (D) Sequence analysis of genomic DNA in WT RAW cells or (E) ΔTAD cells. The latter cells show an in frame deletion of TAD. (F) RT-PCR (mRNA) analysis of WT; KO and ΔTAD cells with WT; TAD or KO specific primers.

Wild type RAW264.7 cells

Genomic DNA

ATGAGCGCCGCGCTTTTCAGCCTGGACAGCCCGGTGCGCGGCACACCCTGGCCCACAGAACCCGCGGCCTTCTAC
GAGCCAGGCAGGGTGGACAAGCCCGGCCGAGGGCCCGAGCCAGGGGATCTGGGGAGCTGGGCTCCACGACTCCT
GCCATGTACGACGACGAGAGCGCCATCGACTTCAGCGCCTACATTGACTCCATGGCCGCCGTGCCCACCCTAGAG
CTGTGCCACGACGAACTCTTCGCCGACCTCTTCAACAGCAACCACAAAGCGGCCGGCGCGGGCGGCCTGGAGCTG
CTGCAGGGCGGCCCTACGCGACCCCCGGGTGTGGGGTCTGTCGCTAGGGGGCCGCTCAAGCGCGAACCCGACTGG
GGCGACGGCGACGCGCCGGGCTCCCTGCTGCCGGCGCAAGTGGCGGTGTGCGCGCAGACAGTGGTGAGCTTGGCG
GCCGCGGCTCAGCCCACTCCACCCACTTCGCCGGAGCCTCCTCGAGGCAGCCCGGGGCCGAGCCTCGCGCCCGGC
ACAGTCCGAGAAAAGGGCGCGGGCAAGAGGGGTCCGGACCGCGGCAGCCCGGAGTACCGGCAGCGGCGCGAGCGC
AACAACATCGCTGTGCGCAAGAGCCGCGACAAGGCCAAGCGCCGCAACCAGGAGATGCAGCAGAAGCTGGTGGAG
TTGTCGGCCGAGAACGAGAAGCTGCATCAGCGCGTGGAGCAGCTCACCCGGGACCTGGCTGGCCTCCGGCAGTTC
TTCAAAAAACTGCCCAGCCCGCCTTTCCTGCCGCCCACCGGCGCCGACTGCCGGTAA

Protein

MSAALFSLDSPVRGTPWPTEPAAFYEPGRVDKPGRGPEPGDLGELGSTTPAMYDDESAIDFSAYIDSMAAVPTLE
LCHDELFADLFNSNHKAAGAGGLELLQGGPTRPPGVGSVARGPLKREPDWGDGDAPGSLLPAQVAVCAQTVVSLA
AAAQPTPPTSPEPPRGSPGPSLAPGTVREKGAGKRGPDRGSPEYRQRRERNNIAVRKSRDKAKRRNQEMQQKLVE
LSAENEKLHQRVEQLTRDLAGLRQFFKKLPSPPFLPPTGADCR

C/EBPδ knock out RAW264.7 cells, clone 1

Genomic DNA

ATGAGCGCCGCGCTTTTCAGCCTGGACAGCCCGGTGCGCGGCACACCCTGGCCCACAGAACCCGCGGCCTTCTAC
GAGCCAGGCAGGGTGGACAAGCCCGGCCGAGGGCCCGAGTGTGGGGTCTGTCGCTAGGGGGCCGCTCAAGCGCGA
ACCCGACTGGGGCGACGGCGACGCGCCGGGCTCCCTGCTGCCGGCGCAAGTGGCGGTGTGCGCGCAGACAGTGGT
GAGCTTGGCGGCCGCGGCTCAGCCCACTCCACCCACTTCGCCGGAGCCTCCTCGAGGCAGCCCGGGGCCGAGCCT
CGCGCCCGGCACAGTCCGAGAAAAGGGC

Protein

MSAALFSLDSPVRGTPWPTEPAAFYEPGRVDKPGRGPECGVCR

C/EBPδ knock out RAW264.7 cells, clone 2

Genomic DNA

ATGAGCGCCGCGCTTTTCAGCCTGGACAGCCCGGTGCGCGGCACACCCTGGCCCACAGAACCCGCGGCCTTCTAC
GAGCCAGGCAGGGTGGACAAGCCCGGCCGAGGGCCCGAGTGGGGTCTGTCGCTAGGGGGCCGCTCAAGCGCGAAC
CCGACTGGGGCGACGGCGACGCGCCGGGCTCCCTGCTGCCGGCGCAAGTGGCGGTGTGCGCGCAGACAGTGGTGA
GCTTGGCGGCCGCGGCTCAGCCCACTCCACCCACTTCGCCGGAGCCTCCTCGAGGCAGCCCGGGGCCGAGCCTCG
CGCCCGGCACAGTCCGAGAAAAGGGC

Protein

MSAALFSLDSPVRGTPWPTEPAAFYEPGRVDKPGRGPEWGLSLGGRSSANPTGATATRRAPCCRRKWRCARRQW

C/EBPδ ΔTAD RAW264.7 cells, clone 1 and 2

Genomic DNA

ATGAGCGCCGCGCTTTTCAGCCTGGACAGCCCGGTGCGCGGCACACCCTGGCCCACAGAACCCGCGGCCTTCTAC
GAGCCAGGCAGGGTGGACAAGCCCGGCCGAGGCCCGGGTGTGGGGTCTGTCGCTAGGGGGCCGCTCAAGCGCGAA
CCCGACTGGGGCGACGGCGACGCGCCGGGCTCCCTGCTGCCGGCGCAAGTGGCGGTGTGCGCGCAGACAGTGGTG
AGCTTGGCGGCCGCGGCTCAGCCCACTCCACCCACTTCGCCGGAGCCTCCTCGAGGCAGCCCGGGGCCGAGCCTC
GCGCCCGGCACAGTCCGAGAAAAGGGCGCGGGCAAGAGGGGTCCGGACCGCGGCAGCCCGGAGTACCGGCAGCGG
CGCGAGCGCAACAACATCGCTGTGCGCAAGAGCCGCGACAAGGCCAAGCGCCGCAACCAGGAGATGCAGCAGAAG
CTGGTGGAGTTGTCGGCCGAGAACGAGAAGCTGCATCAGCGCGTGGAGCAGCTCACCCGGGACCTGGCTGGCCTC
CGGCAGTTCTTCAAAAAACTGCCCAGCCCGCCTTTCCTGCCGCCCACCGGCGCCGACTGCCGGTAA

Protein

MSAALFSLDSPVRGTPWPTEPAAFYEPGRVDKPGRGPGVGSVARGPLKREPDWGDGDAPGSLLPAQVAVCAQTVV
SLAAAAQPTPPTSPEPPRGSPGPSLAPGTVREKGAGKRGPDRGSPEYRQRRERNNIAVRKSRDKAKRRNQEMQQK
LVELSAENEKLHQRVEQLTRDLAGLRQFFKKLPSPPFLPPTGADCR

Figure S2: Genomic DNA and protein sequence of wild type, C/EBPδ knock out and C/EBPδ ΔTAD RAW264.7 clones. The transactivation domain is indicated in red in the wild type sequences.

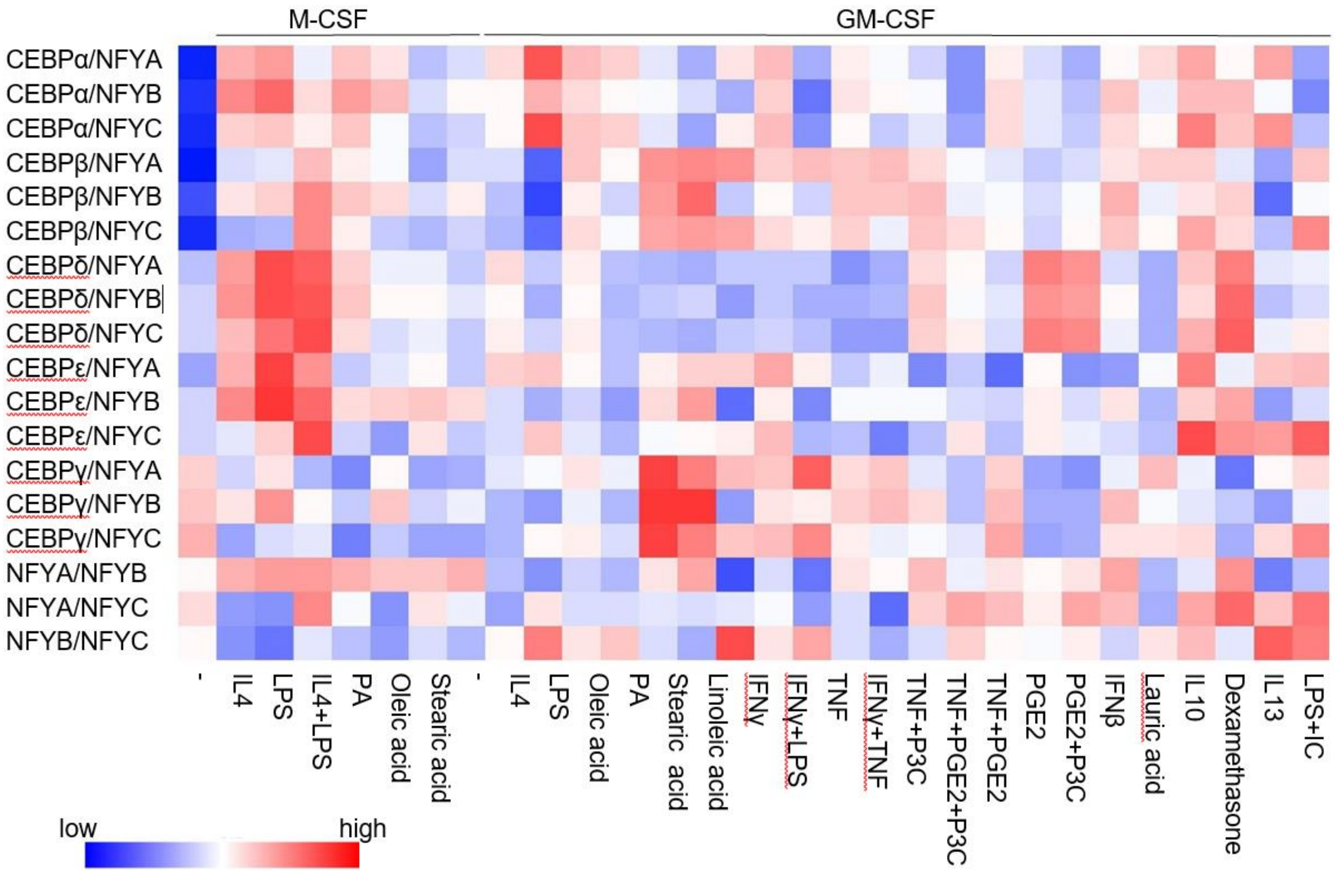

Figure S3. Relative CEBP and NF-Y family member expression in monocytes and macrophages. Heatmap of theexpression ratio of C/EBP and NF-Y family members in monocytes differentiated and activated with different stimuli (data derived from GSE46903).
